# Diagnostic accuracy outcomes of office‐based (outpatient) biopsies in patients with laryngopharyngeal lesions: A systematic review

**DOI:** 10.1111/coa.13897

**Published:** 2022-01-17

**Authors:** Mervyn Owusu‐Ayim, Sushil R. Ranjan, Alison E. Lim, Alexander D. G. Rogers, Jenny Montgomery, Susanne Flach, Jaiganaesh Manickavasagam

**Affiliations:** ^1^ School of Medicine Ninewells Hospital University of Dundee Dundee UK; ^2^ Department of Otolaryngology Head and Neck Surgery Queen Elizabeth University Hospital Glasgow UK; ^3^ Department of Otorhinolaryngology, Head and Neck Surgery Hospital of the University of Munich Munich Germany; ^4^ Department of Otolaryngology Head and Neck Surgery Ninewells Hospital Dundee UK

**Keywords:** flexible endoscopy, In‐office biopsy, laryngopharyngeal lesions, outpatient biopsy, systematic review

## Abstract

**Background:**

In‐office biopsies (IOB) using local anaesthetic for laryngopharyngeal tumours has become an increasingly popular approach since the advent of distal chip endoscopes. Although a wide range of studies advocate use in clinical practice, the widespread application of the procedure is hampered by concerns regarding diagnostic accuracy.

**Objective:**

To assess the diagnostic accuracy of IOB performed via flexible endoscopy. In addition, to analyse modifiable factors that may affect diagnostic accuracy of IOB.

**Design:**

A systematic review following the PRISMA guidelines was conducted. PubMed, EMBASE, the Cochrane Library, Web of Science and CINAHL were used in the literature database search. Quality assessment of included studies was perfomed using the Newcastle‐Ottawa Scale.

**Results:**

A total of 875 studies were identified, 16 of which were included into the systematic review; 1572 successful biopsies were performed using flexible endoscopy; 1283 cases were accurately diagnosed in the outpatient setting (81.6%) and 289 samples did not provide an accurate diagnosis (18.4%). The median sensitivity of IOB was 73%, and the specificity was 96.7%. Analysis of variable factors did not show any significant differences in method of approach, size of equipment (forceps) and additional lighting system or learning curve.

**Conclusion:**

IOB are a viable tool for diagnostic workup of laryngopharyngeal tumours. Clinicians should be wary of reported limitations of IOB when benign or pre‐malignant diagnoses are made. In cases suspicious of malignancy, confirmatory investigation should be conducted.

**Key points**

- In‐office biopsy for laryngopharyngeal tumours has become an increasingly popular approach to acquire laryngopharyngeal samples.
- In office biopsy carries several advantages, some examples including cost‐effectiveness, reduced waiting times and avoidance of general anaesthesia.
- This is the first systematic review to determine diagnostic accuracy outcomes in this patient population.
- IOB are effective tools that can be used to increase efficiency in the diagnostic workup of laryngopharyngeal lesions.

## 1 INTRODUCTION

Efficient allocation of care resources while improving patient outcomes is paramount for any health system. A core part of resource allocation in the patient care pathway involves diagnostic procedures. These are estimated to account for up to 10% of the healthcare budget.^1^ Notably, diagnostic errors when they occur are significant and add substantially to the costs incurred. These costs are greater when considering the psychological impact on a patient when told false positive or false negative results, or prolonged uncertainty.^2^ This stresses the link between long‐term costs attributable to a diagnostic procedure and its diagnostic accuracy, hence the importance of considering these two factors in tandem when adopting a new diagnostic tool.^3^

In the diagnostic workup of laryngopharyngeal tumours, biopsy via direct laryngoscopy (DL) under general anaesthesia (GA) has been recognised as the gold‐standard method of investigation.^4^ The downsides of this include the potential of GA‐associated complications and increased delay between initial presentation and final diagnosis, as theatre suites require advanced booking. Recent progress in flexible endoscopic technology has led to the commendation of lesions being sampled through flexible endoscopes in the in‐office (outpatient) setting.^5^, ^6^ The use of flexible endoscopes for outpatient biopsies (IOB) differs from its traditional counterpart, since patients avoid the need for GA. There is also a reduced incidence of major complications post‐procedure.^7^ Lastly, the speed by which samples can be acquired can reduce the waiting times patients would normally encounter if they were referred for an operative biopsy. This serves as a great advantage to cancer treatment pathways.

Despite this, IOB carry certain drawbacks. Samples acquired from the procedure may be more superficial compared to those conducted in the operative setting, owing to the fact certain anatomical sites are more difficult to reach in the awake patient.^8^ The compliance of patients during the procedure is vital; however, the stressful nature of their hospital visit coupled by anxious thoughts regarding their condition may lead to difficulty in acquiring samples. Furthermore, although GA is avoided, the outpatient setting may not be the ideal place to deal with potential complications.^8^ It is also important to consider that patients who have a negative experience during their IOB, may refuse follow‐up investigations. Lastly, IOB's may require extra appointment time allocation considering the requirements of the procedure, slowing down clinical workflow. Of all these drawbacks, the one which would completely impede the interventions widespread application in clinical practice is diagnostic accuracy.

The question remains whether IOB provide a comparable level of accuracy to operative biopsies under GA. Some authors believe the procedure should be used as an initial work‐up that is followed by direct laryngoscopy, especially in cases where benign/dysplastic diagnoses are made.^6^, ^9^ Others suggest that biopsies identified as malignant can be taken with total confidence therefore demonstrating its role in clinical practice.^10^ Overall, the diagnostic role of IOB can be better established once thorough analysis and critical discussion of variable factors which may affect the accuracy of the intervention has been undertaken.

Apart from the overall sensitivity and specificity of the intervention, modifiable factors which require consideration include the location of lesions, equipment utilised and the biopsy approach used by the surgeon. Firstly, the location of lesions may reduce accuracy considering that certain structures within the laryngopharynx, such as the glottis, may be more difficult to biopsy compared to the pharynx. Postma et al.^11^ demonstrated a 100% diagnostic accuracy when acquiring samples from the pharynx via trans‐nasal oesophagoscopes while Cohen et al.^6^ reported a low sensitivity (69.2%) for biopsies taken from the larynx.

Equipment used may also affect diagnostic accuracy. Alternating the size of forceps may provide better samples and a greater chance of accurate diagnosis. Richards et al.^12^ argued that the type of forceps used for IOB was an important consideration before performing the procedure. Therefore, the size and quality of equipment, the location of lesions as well as the overall approach used by surgeons are factors which can have an impact and as a result require further discussion.

As the use of IOB grows, coupled by the expectation that healthcare interventions should be efficient and cost‐effective, a systematic review seeking to determine the overall diagnostic accuracy of IOB conducted via flexible endoscopy would provide clinicians with an evidence‐based assessment of its strengths and weaknesses. We aim to explore the diagnostic accuracy of in‐office flexible endoscopic biopsy for laryngopharyngeal tumours, while discussing factors which may affect the accuracy of the intervention.

## 2 METHODS

Our systematic review was planned with guidance from the Preferred Reporting Items for Systematic reviews and Meta‐analyses for Protocols (PRISMA‐P).

### 2.1 Review of literature

A systematic search was conducted using MeSH terms and other relevant keywords in the following electronic databases: PubMed, EMBASE, The Cochrane Library, Web of Science and CINAHL. Searches were completed in accordance with guidance from (PRISMA‐P). The final searches were carried out on 17 January 2021. Our search strategy is detailed in Table A1. Table A2 illustrates the inclusion and exclusion criteria used for this study.

The level of evidence for each study was determined according to the guidelines published by the Oxford Centre for Evidence‐based Medicine.^13^

### 2.2 Eligibility criteria

All original research articles with an adult population (>18 years) which reported diagnostic accuracy outcomes from outpatient biopsy were eligible for inclusion. Accuracy outcomes were determined by the following measures: rate of successful biopsies within the outpatient setting, the sensitivity and specificity of IOB. Articles which solely report the use of direct laryngoscopy under GA; investigated children or adolescents were not written in English, conference abstracts or review articles were not eligible for inclusion (Table A2).

### 2.3 Data collection and analysis

Four authors (MOA, SR, SF and JM) independently screened the titles and abstracts of articles identified. Using the criteria from Table A2, identified articles were then scrutinised by authors (MOA, SR) to determine those eligible for inclusion.

Data from the included studies were tabulated in a spreadsheet under the following headings: author name, publication year, evidence level, study design, sample size, type of biopsy method, method of approach, biopsy result, number of accurate versus non‐accurate diagnoses, accurate diagnoses during second attempt (GA direct laryngoscopy).

## 3 RESULTS

### 3.1 Study selection

**FIGURE 1:**
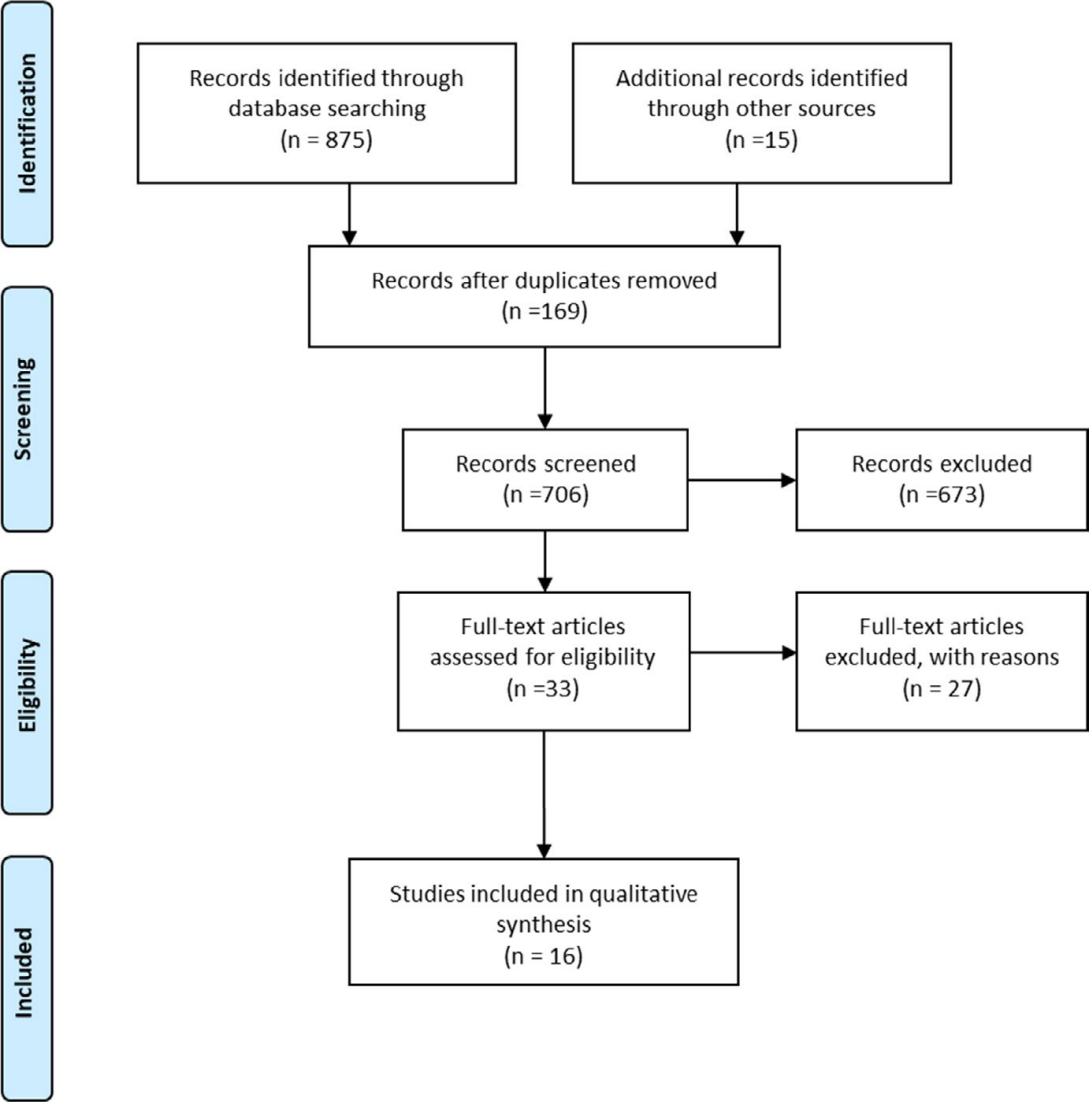
Selection process exhibited by PRISMA flow chart

A total of 875 articles were identified from our database searches, of which 706 were examined for potential eligibility. After screening of titles and abstracts, 33 articles were selected, and full reports of the relevant manuscripts were retrieved; 16 studies met our inclusion criteria and were included in the systematic review (Figure 1). All articles included site of biopsy, type of pathology, number of outpatient biopsies accurately diagnosed and rate of false‐positive versus false‐negative diagnoses.

### 3.2 Study characteristics

A cohort of 1682 patients with laryngopharyngeal lesions were identified from the final eligible articles; 1796 outpatient biopsies were conducted using Flexible Endoscopy in these patients, of which 1572 (87.5%) led to successful tissue acquisition for pathology analysis. The median age of the cohort was 64.4 years (age range: 20–93). Risk of bias assessment was conducted using the Newcastle‐Ottawa scale.^14^ Four of the 16 included studies scored above 6 points, indicating methodological quality (maximum score of 9) (Table A3).

The most common biopsy sites were the larynx 1319 (83.9%), hypopharynx 149 (9.47%), oropharynx 108 (6.87%) and nasopharynx 4 (0.25%); 717 (45.6%) lesions were diagnosed as malignant (squamous cell carcinoma and lymphoma), 117 (7.44%) pre‐malignant (dysplasia, carcinoma in situ) and 454 (28.88%) as benign (polyps, papilloma, etc.).

A total of 1283 (81.6%) outpatient biopsies were accurately diagnosed in the outpatient setting. From data available, 289 (18.4%) samples obtained at outpatient biopsy did not provide an accurate diagnosis. At least 275 patients had an accurate diagnosis made on second attempt, via direct laryngoscopy under GA (*n* = 269) or IOB (*n* = 1) or unspecified procedure (*n* = 5). All repeat procedures yielded accurate diagnoses.

## 4 DISCUSSION

### 4.1 Overview

All 16 studies reported diagnostic accuracy, and the overall calculated accuracy amounted to 81.6% (*n* = 1283; Table A3). Diagnostic accuracy was defined as the percentage of positive cases accurately diagnosed within the outpatient setting, from the total number of biopsies taken. Current literature has described the diagnostic accuracy of IOB to be 81.8% in comparison to DL conducted in the operative setting.^15^ In our study, diagnostic accuracy values ranged between 39.5%^12^ and 93.3%.^16^ Among the false diagnoses, false negatives were far more abundant compared to false positives. In total, 13 studies reported false positives and false negatives. False‐positive and false‐negative results accounted for 1.08% and 13.6%, respectively, of successful biopsy attempts (Table A4).

IOB was well tolerated by patients within the selected studies. Reported side‐effects included coughing or presence of the gag reflex.^16^, ^17^ Only seven (0.41%) patients suffered complications following the procedure, which included epistaxis (*n* = 3),^18^, ^19^ aspiration (*n* = 2),^18^, ^19^ choking (*n* = 1)^20^ and dizziness (*n* = 1).

### 4.2 Sensitivity

Sensitivity is the measure of a test's ability to classify an individual as having a disease.^21^ Of 10 studies that reported sensitivity, the values ranged from 60% to 100% with a median of 73%. Bäck et al.^16^ and Cohen et al.^19^ had the largest cohorts with sensitivity rates included. The latter reported sensitivity of 70.6% while the former reported 100% and 62% depending on whether Narrow‐band Imaging (NBI) was used (100% when used).

### 4.3 Specificity

Specificity is the measure of a test's ability to classify an individual as disease‐free.^21^ Specificity was reported in 10 studies and ranged between 75.6%^20^ and 100%^7^, ^15^, ^18^, ^22^, ^23^ with a median value of 96.7%. It may be relevant that the only study that incorporated trans‐oral flexible endoscopic biopsies not only showed 100% sensitivity^20^ but also reported the lowest specificity rate (75.6%). Whether this reflects a correlative relationship between flexible endoscopic trans‐oral biopsies and a steeper sensitivity‐specificity trade‐off may be relevant for further exploration.

### 4.4 PPV & NPV

Positive predictive value (PPV) is the percentage of patients with a positive test who actually have the disease of interest, whereas negative predictive value (NPV) is the percentage of patients with a negative test who are actually disease‐free.^21^ PPV and NPV were reported from a total of five studies. PPV ranged between 77% ^16^ and 100%^7^, ^15^ with a median value of 93.5%. NPV ranged between 0%^7^ and 100%^16^ with a median of 62%. The outlier value (0%) reported by Naidu et al.^7^ could potentially be explained by their low cohort size (*n* = 11).

### 4.5 Subsite‐specific accuracy

Of the 14 studies that included biopsy subsite information, nine studies reported the larynx as the most common biopsy location. Overall, diagnostic accuracy based on subsite could not be calculated since not all studies reported the relevant numerical data.

Schutte et al.^24^ reported the greatest number of non‐laryngeal biopsy subsites (*n* = 18, 34%). Accuracy rate for their study was reported as 92.5% which was greater than the average accuracy, potentially suggesting that ease of access (i.e. proximal to larynx) may be a contributing factor. It could also be noted that the study by Richards et al.^12^ which reported the lowest diagnostic accuracy predominantly carried out laryngeal biopsies (*n* = 76, 93.8%). However, Afrogeh et al.^23^ who only performed laryngeal biopsies had accuracy of 82.2%, above the overall average and serves as a counter example.

Chang et al.^18^ provided statistical analysis of whether biopsy site could have affected diagnostic accuracy reported by their study. They found no statistical significance between biopsy location and accuracy (χ = 6.30, *p* = .614). Additional studies may be required to assess the relationship between IOB sub‐site and accuracy.

### 4.6 Technique

A total of 13 studies conducted IOB via a trans‐nasal approach. Two studies^17^, ^20^ studied trans‐oral biopsy. There was no reported difference in tolerability between biopsies conducted trans‐nasally versus trans‐orally. Of the studies that conducted trans‐oral biopsies, a significant difference in sensitivity/specificity levels compared to trans‐nasal biopsies were not identified. It remains difficult to ascertain whether trans‐oral biopsies grant greater accuracy compared to trans‐nasal biopsies. Future comparative research would help to answer this question.

### 4.7 Additional lighting system

Bäck et al.^16^ split their cohort into two groups, one with IOB patients using the NBI filter and the other with white light high‐definition TV (WLHD). They reported higher sensitivity and specificity rates for the NBI group (100% and 84% respectively) compared to the WLHD group (62% and 81%). Chang et al.^18^ also utilised NBI with their IOB and reported sensitivity rate of 97.2% and specificity rate of 100%, which were well above the overall average for the 16 studies. Based on these two studies, it is likely that using NBI could be a useful addition when performing IOB. Prospective randomised trials could be carried out to formally analyse the effect of NBIs and other lighting systems on the diagnostic accuracy of IOB.

### 4.8 Forceps size

Ten studies reported the endoscopic system used for biopsies as well as the size of biopsy forceps.^12^, ^15^, ^18^, ^19^, ^20^, ^22^, ^24^, ^25^, ^26^, ^27^ The most common endoscopes included those made by KayPentax, Medtronic and Olympus (Table A5). Most authors opted for 1.8‐mm or 2‐mm forceps (serrated or non‐serrated). Studies that chose biopsy forceps greater than 2 mm^6^, ^8^, ^20^, ^26^ were found to have an average diagnostic accuracy of 73.9% while studies that chose biopsy forceps <2 mm had a diagnostic accuracy of 74.6%.^12^, ^15^, ^22^, ^24^, ^25^, ^27^ The significance of this result remains difficult to determine as studies were conducted with different methodologies and varying cohorts. In addition, factors such as patient compliance would have an equal or greater effect on size of sample acquired, possibly affecting diagnostic accuracy. It is difficult to make a fair comparison based on these results alone, whether biopsy forceps size correlates to improved diagnostic accuracy. Future studies comparing these components are needed.

### 4.9 Learning curve

Chang et al.^18^ split up their IOB procedures into two groups (initial 30 procedures and last 60 procedures) to analyse whether surgeon experience was associated with accuracy. They reported that experience with IOB did not show statistically significant association with diagnostic accuracy (*p* = .131). This result may need to be taken cautiously as inexperience could increase the risk of misdiagnosis or procedure‐related harm to patients. Furthermore, Chang et al.^18^ had assessed if the learning curve of a “single experienced laryngologist” affected diagnostic accuracy, bringing into question if this finding could be generalised to the wider surgeon population. Chang et al.^18^ also reported a diagnostic accuracy of 98.9% with only one incorrect diagnosis among the 90 procedures. Hence, accuracy of the initial 30 and subsequent 60 procedures would not have differed much, potentially resulting in an under‐estimate of the role of experience with IOB.

Schutte et al.,^24^ Richards et al.^12^ and Bäck et al.^16^ commented that experienced surgeons performed the IOB in their studies. However, the association of experience with diagnostic accuracy was not reported in these studies. The diagnostic accuracies reported by them were far apart, ranging from 39.5%^12^ to 93.3%.^16^ However, without information about surgeon experience from the other studies it was not possible to quantify its association with diagnostic accuracy.

### 4.10 Repeat biopsies

All repeat biopsies yielded accurate diagnoses and almost all of them were direct laryngoscopies (*n* = 269/275). In general, the studies considered direct laryngoscopy under general anaesthesia as the gold‐standard procedure against which IOB were evaluated.

Direct laryngoscopy re‐biopsies in the studies reporting a 100% diagnostic accuracy could be due to an element of cautious deliberation. The majority of re‐biopsies were performed for a high clinical suspicion of malignancy but negative initial IOB result. Repeat procedures would likely involve increased care in tissue sampling selection, potentially better access and greater total volume of tissue volume biopsied.

Schutte et al. was the only group that carried out a repeat IOB among their re‐biopsies (one IOB, one direct laryngoscopy).^24^ They obtained an accurate diagnosis with the repeat IOB but did not state why that patient was selected for it as opposed to a direct laryngoscopy. The sample size of patients undergoing IOB re‐biopsy (*n* = 1) is too small to evaluate the usefulness of repeat IOB. This limitation presents an area that could be further explored in future studies.

### 4.11 Significance of findings

Our results indicate that IOB can be a highly useful tool in the diagnostic workup pathway. 87.5% of performed biopsies were successful in acquiring adequate tissue for pathology analysis. Accurate diagnosis was achieved in 81.6% of cases. IOB appear to offer advantages such as timely results, cost savings compared to operative biopsies. Farias et al.^15^ reported potential annual savings of $50 140.80 if IOB were used as a primary method of investigation. Similarly, Lippert et al. reported a reduced duration in waiting time with successful biopsies.^15^, ^27^ Furthermore, IOB carry an effective diagnostic accuracy based on reported NPV and sensitivities within our included studies. However, a high level of suspicion is required when biopsies return as negative considering the false‐negative rates within our studies. It is advised that future management decisions in such cases should be taken in conjunction with clinical findings and additional information available to the clinicians. We believe it is necessary to assess the validity of results received from an IOB, in the context of the patient. In cases of doubt, confirmatory investigation via direct laryngoscopy can be conducted.

### 4.12 Limitations

Limitations of our review include the inclusion of retrospective cohort studies. This makes studies included more prone to biases such as publication and ascertainment bias. Our systematic review also excluded non‐English primary articles and, hence, relevant papers in other languages may have been missed.

Studies included in this review did not have patient cohorts that were well‐matched based on factors that could affect diagnostic accuracy such as lesion attributes (e.g. tumour size, exophytic vs. endophytic) and site of biopsy. With biopsy site, for example, more accessible parts of the laryngopharynx (e.g. tongue base) would be expected to be easier to obtain specimens from and possibly be associated with better diagnostic accuracy outcomes. Exophytic lesions may also confer an advantage with regards to obtaining better biopsy samples (hence, leading to high accuracy rates) compared to ulcerative endophytic lesions.

## 5 FUTURE PROSPECTS

Research on the differences between transnasal approach and transoral approach and their associations with diagnostic accuracy is an area that could be further studied. Prospective studies could also be undertaken with randomisation to allow for an analysis of higher quality evidence. Modifiable variables such as type of forceps (e.g. serrated) and lighting systems (e.g. NBI) could also be individually studied to further determine their impact on diagnostic accuracy allowing clinicians to utilise the benefits of IOB while continuing to minimise its limitations.

## 6 CONCLUSION

In conclusion, IOB is an effective tool for the diagnostic workup of laryngopharyngeal lesions. Its advantages would be beneficial to clinicians who are seeking to reduce procedural time and improve on cost savings. Available evidence suggests clinicians should remain cautious when interpretating negative biopsy results, especially in the context of strong suspicion and should conduct confirmatory testing in such scenarios.

## AUTHOR CONTRIBUTIONS

Mervyn Owusu‐Ayim and Sushil R Ranjan should be considered joint first authors. Both contributed to conception and design of study, acquisition of data and analysis, drafting and revising the manuscript. Alison E Lim and Alexander D G Rogers were involved in analysis of data and revising the manuscript. Jenny Montgomery, Susanne Flach and Jaiganesh Manickavasagam contributed a supervisory role, reviewing and revising the manuscript, as well as aiding in the analysis and interpretation of data.

## ETHICAL CONSIDERATIONS

This study did not require ethical approval as there were no patient‐identifiable data included. This study was registered with the international prospective systematic review register (PROSPERO: CRD42019158186).

## Data Availability

The data that supports the findings of this study are available in the supplementary material of this article.

## APPENDIX 1

**TABLE A1:** Search strategy

Search strategy	("larynx" or "laryngeal" or "laryngo*" or "pharyngeal" or "pharynx" or "oesophageal" or "oesophagus") and ("tumour" or "lesion" or "benign" or "malign*" or "carcinoma" or "dysplasia") AND ("biopsy" or "biopsies") AND ("office" OR "outpatient" or "in‐office" or "office‐based")

**TABLE A2:** Inclusion and exclusion criteria

PICOS strategy	Inclusion criteria	Exclusion criteria
P‐Population	Patients ≥18 years of age, with pharyngeal or laryngeal benign or (pre‐)malignant lesions.	Children and adolescents
I‐Intervention	Transnasal Flexible Endoscopic biopsy for a laryngopharyngeal lesion performed in the outpatient setting under local anaesthetic.	Any other method of biopsy for laryngopharyngeal lesion conducted under general anaesthetic.
C‐Comparison	Patients who have received biopsy under general anaesthetic for a laryngopharyngeal lesion.	
O‐Outcome	Accuracy (rate of successful biopsies, sensitivity and specificity)	Studies that do not provide data on this outcome.

**TABLE A3:** Studies Included in systematic review

Paper title	Paper authors	Publication type	Evidence type and level	Sample size	Type of study	Newcastle‐Ottawa Scale (Max score = 9)
The feasibility of NBI in patients with suspected upper airway lesions: A multicenter study^16^	Leif J Bäck 1, Jami Rekola 2, Lassi Raittinen 3, Elina Halme 3, Petra Pietarinen 1, Harri Keski‐Säntti 1, Leena‐Maija Aaltonen 1, Antti A Mäkitie 1, Antti Raappana 4, Jukka Tikanto 4, Aleksi Schrey 2, Reidar Grenman 2, Jussi Laranne 3, Petri Koivunen 4, Heikki Irjala 2	Full journal article	Level II	125	Cohort studies	7
Reliability of office‐based narrow‐band imaging‐guided flexible laryngoscopic tissue samplings^18^	Jacob T. Cohen, MD; Ahmad Safadi, MD; Dan M. Fliss, MD; et al	Full journal article	Level IV	102	Cohort studies	4
Office‐based biopsies for laryngeal lesions: Analysis of consecutive 581 cases^26^	Cha W., Yoon B.‐W., Jang J.Y., Lee J.C., Lee B.J., Wang S.‐G., Cho J.K., Cho I.	Full journal article	Level IV	581	Cohort studies	4
Transnasal flexible fiberoptic in‐office laryngeal biopsies‐our experience with 117 patients with suspicious lesions.^19^	Jacob T. Cohen, M.D.* and Limor Benyamini, M.D.	Full journal article	Level IV	117	Cohort studies	7
Transoral flexible laryngoscope biopsy: Safety and accuracy^20^	Nabeel Humayun Hassan Rahila Usman Muhammad Yousuf Ahmad Nawaz Ahmad Ismail Hirani	Full journal article	Level IV	47	Cohort studies	4
Application of flexible endoscopy‐based biopsy in the diagnosis of tumour pathologies in otorhinolaryngology^22^	Saga, Carlos	Full journal article	Level IV	30	Cohort studies	4
The utility of office‐based biopsy for laryngopharyngeal lesions: Comparison with surgical evaluation^12^	Amanda L Richards 1, Manikandan Sugumaran, Jonathan E Aviv, Peak Woo, Kenneth W Altman	Full journal article	Level IV	76	Cohort studies	4
Digital video laryngoscopy and flexible endoscopic biopsies as an alternative diagnostic workup in laryngopharyngeal cancer: A prospective clinical study.^24^	Schutte H.W., Takes R.P., Slootweg P.J., Arts M.J.P.A., Honings J., van den Hoogen F.J.A., Marres H.A.M., van den Broek G.B.	Full journal article	Level III	53	Cohort studies	7
In‐office cup biopsy and laryngeal cytology versus operating room biopsy for the diagnosis of pharyngolaryngeal tumors: Efficacy and cost‐effectiveness^15^	Farías F.C., Cobeta I., Souviron R., Barberá R., Mora E., Benito A., Royuela A.	Full journal article	Level II	88	Randomised control trial	6
Office‐based transnasal esophagoscopy biopsies for histological diagnosis of head and neck patients^27^	Mohammed H., Del Pero M., Coates M., Masterson L., Tassone P., Burrows S., Nassif R.	Full journal article	Level IV	121	Cohort studies	3
Reliability of office‐based narrow‐band imaging‐guided flexible laryngoscopic tissue samplings^18^	Chang, C; Lin, WN; Hsin, LJ; Lee, LA; Lin, CY; Li, HY; Liao, CT; Fang, TJ	Full journal article	Level IV	90	Case control	3
Application of liquid‐based transepithelial flexible brush cytology in the detection of high‐grade laryngeal mucosal lesions.^23^	Afrogheh A., Pelser A., Hille J., Attwood R., Loock J., Schubert P.T.	Full journal article	Level IV	50	Cohort studies	6
Comparison of efficacy, safety, and cost‐effectiveness of in‐office cup forcep biopsies versus operating room biopsies for laryngopharyngeal tumors^7^	Naidu H., Noordzij J.P., Samim A., Jalisi S., Grillone G.A.	Full journal article	Level IV	12	Cohort studies	7
Comparison of trans‐nasal laryngoscopic office based biopsy of laryngopharyngeal lesions with traditional operative biopsy^28^	Zalvan C.H., Brown D.J., Oiseth S.J., Roark R.M.	Full journal article	Level IV	26	Cohort studies	4
Cost analysis of channeled, distal chip laryngoscope for in‐office laryngopharyngeal biopsies^25^	Marcus S., Timen M., Dion G.R., Fritz M.A., Branski R.C., Amin M.R.	Full journal article	Level IV	48	Cohort Studies	4
In‐office biopsy of upper airway lesions: Safety, tolerance, and effect on time to treatment^17^	Lippert D., Hoffman M.R., Dang P., McCulloch T.M., Hartig G.K., Dailey S.H.	Full journal article	Level IV	116	Cohort studies	3

**TABLE A4:** Accuracy outcome data

Paper title	Paper authors	Biopsy successful using FEB (tissue acquisition)	Biopsy non‐successful using FEB (inadequate tissue)	Sensitivity	Specificity	Number accurately diagnosed in outpatient	Number not accurately diagnosed in outpatient	False positive	False negative	Diagnostic accuracy
The feasibility of NBI in patients with suspected upper airway lesions: A multicenter study^16^	Leif J Bäck 1, Jami Rekola 2, Lassi Raittinen 3, Elina Halme 3, Petra Pietarinen 1, Harri Keski‐Säntti 1, Leena‐Maija Aaltonen 1, Antti A Mäkitie 1, Antti Raappana 4, Jukka Tikanto 4, Aleksi Schrey 2, Reidar Grenman 2, Jussi Laranne 3, Petri Koivunen 4, Heikki Irjala 2	85				61				71.8%
Reliability of office‐based narrow‐band imaging‐guided flexible laryngoscopic tissue samplings^18^	Jacob T. Cohen, MD; Ahmad Safadi, MD; Dan M. Fliss, MD; et al	96	6	70.6	96.7	65	31	1	30	67.7%
Office‐based biopsies for laryngeal lesions: Analysis of consecutive 581 cases^26^	Cha W., Yoon B.‐W., Jang J.Y., Lee J.C., Lee B.J., Wang S.‐G., Cho J.K., Cho I.	576	5	Malignancy: 78.2 Malignancy/Pre‐malginancy: 88.2	Malignancy: 100% Malignancy/Pre‐malignancy: 86.7	526	50		50	91.3%
Transnasal flexible fiberoptic in‐office laryngeal biopsies‐our experience with 117 patients with suspicious lesions.^19^	Jacob T. Cohen, M.D.* and Limor Benyamini, M.D.	110	7	70.6	96.7	71	33	1	32	64.5%
Transoral flexible laryngoscope biopsy: Safety and accuracy^20^	Nabeel Humayun Hassan Rahila Usman Muhammad Yousuf Ahmad Nawaz Ahmad Ismail Hirani	43		75.6	100	31	10		10	72.1%
Application of flexible endoscopy‐based biopsy in the diagnosis of tumour pathologies in otorhinolaryngology^22^	Saga, Carlos	29		73	100	23	7		7	79.3%
The utility of office‐based biopsy for laryngopharyngeal lesions: Comparison with surgical evaluation^12^	Amanda L Richards 1, Manikandan Sugumaran, Jonathan E Aviv, Peak Woo, Kenneth W Altman	81	4	60	87	32	17			39.5%
Digital video laryngoscopy and flexible endoscopic biopsies as an alternative diagnostic workup in laryngopharyngeal cancer: A prospective clinical study.^24^	Schutte H.W., Takes R.P., Slootweg P.J., Arts M.J.P.A., Honings J., van den Hoogen F.J.A., Marres H.A.M., van den Broek G.B.	53		PPV 100%	NPV: 33%	49	4			92.5%
In‐office cup biopsy and laryngeal cytology versus operating room biopsy for the diagnosis of pharyngolaryngeal tumors: Efficacy and cost‐effectiveness^15^	Farías F.C., Cobeta I., Souviron R., Barberá R., Mora E., Benito A., Royuela A.	88		81.1	100	72	16		16	81.8%
Office‐based transnasal esophagoscopy biopsies for histological diagnosis of head and neck patients^27^	Mohammed H., Del Pero M., Coates M., Masterson L., Tassone P., Burrows S., Nassif R.	115	19	N/A	N/A	107	6		6	93.0%
Reliability of office‐based narrow‐band imaging‐guided flexible laryngoscopic tissue samplings^18^	Chang, C; Lin, WN; Hsin, LJ; Lee, LA; Lin, CY; Li, HY; Liao, CT; Fang, TJ	90		97.2	100	84	6	5	1	93.3%
Application of liquid‐based transepithelial flexible brush cytology in the detection of high‐grade laryngeal mucosal lesions.^23^	Afrogheh A., Pelser A., Hille J., Attwood R., Loock J., Schubert P.T.	46	4	77.1	100	38	8	0	8	82.6%
Comparison of efficacy, safety, and cost‐effectiveness of in‐office cup forcep biopsies versus operating room biopsies for laryngopharyngeal tumors^7^	Naidu H., Noordzij J.P., Samim A., Jalisi S., Grillone G.A.	11				7	4		4	63.6%
Comparison of trans‐nasal laryngoscopic office based biopsy of laryngopharyngeal lesions with traditional operative biopsy^28^	Zalvan C.H., Brown D.J., Oiseth S.J., Roark R.M.	26				21	5	3	2	80.8%
Cost analysis of channeled, distal chip laryngoscope for in‐office laryngopharyngeal biopsies^25^	Marcus S., Timen M., Dion G.R., Fritz M.A., Branski R.C., Amin M.R.	26	2	N/A	N/A	16				61.5%
In‐office biopsy of upper airway lesions: Safety, tolerance, and effect on time to treatment^17^	Lippert D., Hoffman M.R., Dang P., McCulloch T.M., Hartig G.K., Dailey S.H.	97	17			80	17		17	82.5%

**TABLE A5:** Endoscopic equipment used in studies

Paper title	Paper authors	Type of Endoscope	Biopsy forceps (type)	Biopsy forceps size
Reliability of office‐based narrow‐band imaging‐guided flexible laryngoscopic tissue samplings^18^	Jacob T. Cohen, MD; Ahmad Safadi, MD; Dan M. Fliss, MD; et al	FNL10RP3; KayPentax or ENT 2000; Vision Sciences	Laryngeal biopsy forceps; Medtronic	2 mm diameter
Office‐based biopsies for laryngeal lesions: Analysis of consecutive 581 cases^26^	Cha W., Yoon B.‐W., Jang J.Y., Lee J.C., Lee B.J., Wang S.‐G., Cho J.K., Cho I.	Flexible endoscope (ENF‐VT, Olympus Corporation, Tokyo, Japan)	2‐mm EndoSheath channel and reusable fenestrated round‐cup biopsy forceps (FB‐19SX‐1)	2‐mm
Transnasal flexible fiberoptic in‐office laryngeal biopsies‐our experience with 117 patients with suspicious lesions.^19^	Jacob T. Cohen, M.D.* and Limor Benyamini, M.D.	Pentax‐FNL‐10 RP3	Laryngeal Biopsy Forceps, Medtronic, Minneapolis, MN, USA	2‐mm
Transoral flexible laryngoscope biopsy: Safety and accuracy^20^	Nabeel Humayun Hassan Rahila Usman Muhammad Yousuf Ahmad Nawaz Ahmad Ismail Hirani	Olympus made scope, BF type TE2	N/A	2.8 mm
Application of flexible endoscopy‐based biopsy in the diagnosis of tumour pathologies in otorhinolaryngology^22^	Saga, Carlos	Olympus BF 240 and Olympus Evis Exera III Cv‐190, both with a working channel	Olympus Endojaw FB 231D	1.9 mm
The utility of office‐based biopsy for laryngopharyngeal lesions: Comparison with surgical evaluation^12^	Amanda L Richards 1, Manikandan Sugumaran, Jonathan E Aviv, Peak Woo, Kenneth W Altman	ENT‐5000, Vision Sciences, Inc. or VNL‐1570STK, KayPENTAX Montvale, NJ	2‐mm channel endosheath and 1.8‐mm non‐serrated cup biopsy forceps	1.8 mm non‐serrated cup biopsy forceps
Digital video laryngoscopy and flexible endoscopic biopsies as an alternative diagnostic workup in laryngopharyngeal cancer: A prospective clinical study.^24^	Schutte H.W., Takes R.P., Slootweg P.J., Arts M.J.P.A., Honings J., van den Hoogen F.J.A., Marres H.A.M., van den Broek G.B.	VNL‐1570STK; Pentax, Tokyo, Japan	Radial Jaw 4, pulmonary standard capacity with needle, standard 2.0 mm; Boston Scientific, Heredia, Costa Rica	1.8 mm diameter forceps with needle
In‐office cup biopsy and laryngeal cytology versus operating room biopsy for the diagnosis of pharyngolaryngeal tumors: Efficacy and cost‐effectiveness^15^	Farías F.C., Cobeta I., Souviron R., Barberá R., Mora E., Benito A., Royuela A.	Aso‐fiberscope with working channel (K. Storz, model 11001 KL)	microforceps (K. Storz, model 11001 UD)	1.8 mm
Office‐based transnasal esophagoscopy biopsies for histological diagnosis of head and neck patients^27^	Mohammed H., Del Pero M., Coates M., Masterson L., Tassone P., Burrows S., Nassif R.	Pentax 80 K Series Digital Video Endoscope; Pentax, Slough, UK	Pentax KW1811S 1.8‐mm flexible biopsy forceps; Pentax	1.8 mm
Cost analysis of channeled, distal chip laryngoscope for in‐office laryngopharyngeal biopsies^25^	Marcus S., Timen M., Dion G.R., Fritz M.A., Branski R.C., Amin M.R.	Video Naso‐Pharyngo‐Laryngoscope VNL‐1570STK, Pentax Medical, Montvale, NJ	1.8‐mm non‐serrated cup biopsy forceps.	1.8 mm
